# A MAPS Vaccine Induces Multipronged Systemic and Tissue-Resident Cellular Responses and Protects Mice against Mycobacterium tuberculosis

**DOI:** 10.1128/mbio.03611-22

**Published:** 2023-02-07

**Authors:** Joanne M. O’Hara, Shoko Wakabayashi, Noman Siddiqi, Elaine Cheung, Gregory H. Babunovic, Claudette M. Thompson, Ying-Jie Lu, Eric J. Rubin, Richard Malley, Fan Zhang

**Affiliations:** a Division of Infectious Diseases, Boston Children’s Hospital, Boston, Massachusetts, USA; b Harvard Medical School, Boston, Massachusetts, USA; c Department of Immunology and Infectious Diseases, Harvard T.H. Chan School of Public Health, Boston, Massachusetts, USA; d Program in the Biological and Biomedical Sciences, Harvard University, Boston, Massachusetts, USA; Max Planck Institute for Infection Biology

**Keywords:** B cell, *Mycobacterium tuberculosis*, T cell, tuberculosis, vaccine

## Abstract

Tuberculosis (TB) remains a leading cause of morbidity and mortality worldwide. To date, the mainstay of vaccination involves the use of Mycobacterium bovis bacillus Calmette-Guérin (BCG), a live-attenuated vaccine that confers protection against extrapulmonary disease in infants and children but not against lung disease. Thus, there is an urgent need for novel vaccines. Here, we show that a multicomponent acellular vaccine (TB-MAPS) induces robust antibody responses and long-lived systemic and tissue-resident memory Th1, Th17, and cytotoxic CD4^+^ and CD8^+^ T cells, and promotes trained innate immunity mediated by γδT and NKT cells in mice. When tested in a mouse aerosol infection model, TB-MAPS significantly reduced bacterial loads in the lungs and spleens to the same extent as BCG. When used in conjunction with BCG, TB-MAPS further enhanced BCG-mediated protection, especially in the lungs, further supporting this construct as a promising TB vaccine candidate.

## INTRODUCTION

Tuberculosis (TB), the disease caused by Mycobacterium tuberculosis (Mtb) infection, is a leading cause of morbidity and mortality worldwide. In 2021, there were approximately 10.6 million TB cases (including over 1.5 million children) and 1.5 million TB-related deaths (including 0.2 million patients with HIV coinfection). In addition, multidrug-resistant Mtb is on the rise, posing even greater threats to public health (1). In 2014, WHO passed the “End TB Strategy,” aiming to “reduce TB incidence by 80%, TB death by 90% and eliminate catastrophic costs of TB-affected households by 2030” and stating that a new and effective vaccine would be required to meet these goals. However, despite global efforts, TB incidence declined by only 9% cumulatively between 2015 and 2019, far from the milestone of a 20% reduction by 2020. Hence, more effective approaches to combat TB, such as preventive vaccination, are in urgent need.

Since its introduction in 1921, Mycobacterium bovis bacillus Calmette-Guérin (BCG) remains the only licensed TB vaccine to date. BCG is widely used in countries with a high prevalence of TB and demonstrates partial protection against TB meningitis and other forms of disseminated TB in children. More recently, BCG was shown to reduce the rate of sustained QuantiFERON conversion, a marker of sustained Mtb infection, in a high-transmission setting (2). Importantly, however, the main role of BCG is to prevent extrapulmonary infection (3, 4), with far less impact on pulmonary primary or reactivated disease, which accounts for a significant portion of TB transmission in the community. Thus, novel vaccines that can prevent and/or reduce Mtb pulmonary infection and disease are urgently needed.

While many vaccines, for TB and other diseases, have been designed empirically, a deeper understanding of the nature of protective immunity may assist with successful vaccine development. In the case of Mtb, mechanisms of protection have not been firmly established, although a combination of Th1 cells, CD8^+^ T cells (5–9), and, to a lesser extent, humoral responses (10, 11) have long been hypothesized to play important roles. Accordingly, current efforts in vaccine development, with 14 vaccine candidates at different stages of clinical testing (including whole-cell based vaccines [WCV], virus-vectored vaccines, or acellular [subunit] vaccines) (12, 13), have focused on generating Th1 and cytotoxic CD8 responses. However, recent studies in mice, nonhuman primates (NHP), and humans suggest that anti-Mtb immunity may benefit from activities of other T cell populations, including Th17 cells and nonconventional T cells, such as γδT cells and natural killer T (NKT) cells. Th17 cells have been shown to contribute to protection in two different ways: facilitating Th1-mediated defense by recruiting functional Th1 cells to the infected lung tissue (14, 15) or mediating protection entirely independent of gamma interferon (IFN-γ)/Th1 responses (16). Balancing Th1/Th17 immunity may be critical to provide optimal protection and not induce pathology (17, 18). Moreover, evidence shows that IL-17 production by lung γδT cells in response to Mtb infection can promote the formation and maturation of granuloma (19, 20) and subsequent sequestration and killing of Mtb (21, 22). Despite their distinct T cell receptor (TCR)/activation mechanism, γδT cells could develop a memory-like phenotype (so-called trained innate immunity) and play a role in recall responses during infection (23–25). Another arm of trained innate immunity is NKT cells, which have been shown to mediate the killing of intracellular Mtb in infected cells and provide protection in mice and nonhuman primates (NHP) (26–28). Furthermore, studies suggest that in addition to systemic responses (mediated by T cells traveling primarily between circulation and lymphoid tissues), generating tissue-resident memory/memory-like T cells may be critical in protection against local Mtb infections (such as pulmonary infection) (29–31). Taken together, these findings point out the importance of generating multipronged cellular responses to Mtb for more effective protection against this pathogen.

We have developed a subunit vaccine platform called the Multiple Antigen Presenting System (MAPS) (32). Using the MAPS technology, we can generate highly immunogenic antigen complexes that induce robust humoral and cellular responses and thus provide multifaceted protection against the target pathogen (32, 33). This vaccine platform relies on the high-affinity association of biotin with rhizavidin and enables the formation of polysaccharide-protein complexes, whereby the polysaccharide is biotinylated and pathogen-specific proteins of interest are genetically fused to rhizavidin. We have previously shown that MAPS complexes generate robust T cell (including CD4^+^ Th1/Th17 and CD8^+^) and antibody responses to both protein and polysaccharide components. The MAPS platform has been used to target several pathogens for which a combination of antibodies to polysaccharides, antibodies to proteins, or T cell responses may be beneficial, including pneumococcus (32), Staphylococcus aureus (33), Salmonella enterica serovar Typhi and serovar Paratyphi (34), and SARS-CoV-2 (35). A 24-valent MAPS pneumococcal vaccine has successfully completed phase 2 trials in adults (36) and is currently being evaluated in infants.

Here, we designed and evaluated an experimental TB-MAPS vaccine comprising up to 7 Mtb protein antigens (Table 1). We demonstrate that immunization with TB-MAPS induces potent antibodies and cellular responses to the target Mtb antigens and protects mice against Mtb aerosol infection to the same extent as with BCG. Moreover, we show that TB-MAPS, when used in conjunction with BCG, significantly enhances protection compared to either vaccine alone, especially against Mtb lung infection. Finally, we evaluate the differential role of the interleukin-12 p40 (IL-12p40) signaling pathway in TB-MAPS-mediated protection against local Mtb infection in the lungs or dissemination in the blood.

**TABLE 1:** Composition of fusion proteins in MAPS complexes

Vaccine	Composition
MAPS1	BCPS1-lipidated rhavi
	BCPS1-rhavi-ESAT6/CFP10
	BCPS1-rhavi-TB9.8/TB10.4
	BCPS1-rhavi-MPT64
	BCPS1-rhavi-MPT83

MAPS2	BCPS1-lipidated rhavi
	BCPS1-rhavi-TB9.8/TB10.4-MPT83
	BCPS1-rhavi-ESAT6/CFP10-MPT64
	BCPS1-rhavi-MPT51

## RESULTS

### TB-MAPS elicits robust antigen-specific antibody and cellular responses and protects mice against Mtb infection.

**FIG 1:**
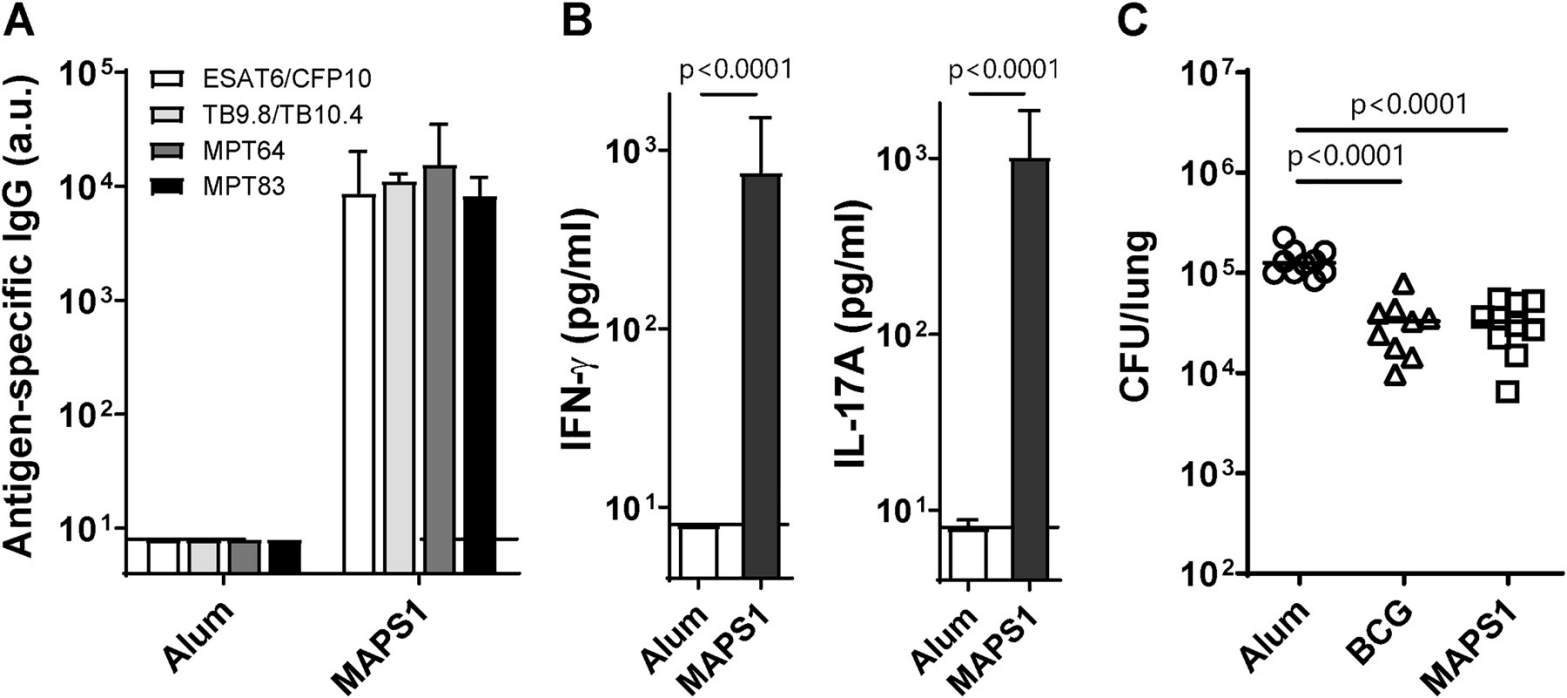
Immunization with MAPS1 elicits robust Mtb antigen-specific antibody and cellular responses and protects mice against Mtb aerosol infection. (A and B) Mice (*n* = 10 per group) were immunized three times with adjuvant vehicle (Alum) or MAPS1 (MAPS1). Peripheral blood was taken 2 weeks after the last immunization for antibody and cellular response analysis. (A) IgG titers against each Mtb protein. Titers were expressed in arbitrary units (a.u.) compared to a standard serum for each Mtb protein. (B) IFN-γ and IL-17A levels in culture supernatants after stimulating blood cells with a mixture of six Mtb proteins (2 μg/mL). (C) Mice (*n* = 10 per group) were immunized three times with adjuvant alone (Alum) or MAPS1 as described above. The BCG group received one dose of BCG at the first immunization and no boosters. Two weeks after the third immunization (6 weeks after BCG immunization), mice were infected with aerosolized Mtb strain H37Rv. One month later, lungs were harvested for CFU enumeration. Bars indicate medians plus 95% confidence intervals. Lines indicate medians. Dotted lines represent the lower detection limit.

TB-MAPS complexes were prepared by affinity coupling rhizavidin (rhavi)-Mtb fusion antigens to the biotinylated pneumococcal type 1 capsular polysaccharide (BCPS1). The first formulation of TB-MAPS contained 6 Mtb antigens (ESAT6, CFP10, TB9.8, TB10.4, MPT64, and MPT83) and lipidated rhavi, a Toll-like receptor 2 (TLR2) agonist that can further enhance MAPS-induced cellular responses (32, 37) (Table 1, MAPS1). These antigens were selected based on possible protective roles as reported in the literature and the ability to be expressed and purified as fusion proteins with rhizavidin. Mice received three subcutaneous immunizations of MAPS1, and a negative-control group received adjuvant alone (Alum). Vaccination with MAPS1 induced high-titer IgG directed against all six Mtb antigens (Fig. 1A). MAPS-induced antigen-specific cellular responses were evaluated by *ex vivo* stimulation of heparinized peripheral blood with purified Mtb proteins. We had previously demonstrated (32) that immunization with MAPS constructs containing Mtb antigens generates cellular responses to the individual antigens. Here, to limit the amount of blood required from mice, we stimulated the peripheral blood samples with a mixture of the antigens. Upon stimulation, blood cells from MAPS1-immunized mice but not from control mice produced IFN-γ and IL-17A (Fig. 1B). The efficacy of the MAPS1 vaccine was further evaluated in an Mtb aerosol infection model, using BCG as a positive control. Mice were infected with Mtb strain H37Rv 3 weeks after the third immunization. One month after infection, lungs were harvested, homogenized, and plated for CFU enumeration. Compared to the negative-control mice that had an average of 10^5^ CFU (median and 95% confidence interval [CI], 125,676 [99,730, 165,000]) in their lungs postinfection (Fig. 1C, Alum), mice that received either BCG (median and 95% CI, 32,838 [14,338, 43,784]) or MAPS1 (median and 95% CI, 32,635 [15,000, 52,297]) had significantly reduced bacterial burden in the lung tissues (*P* < 0.0001 for both groups) (Fig. 1C, BCG and MAPS1). The levels of protection mediated by BCG and MAPS1 were comparable: both led to about a 4-fold reduction of lung CFU compared to the control group.

### Combined vaccination with BCG and TB-MAPS results in significantly enhanced protection compared to either vaccine alone.

**FIG 2:**
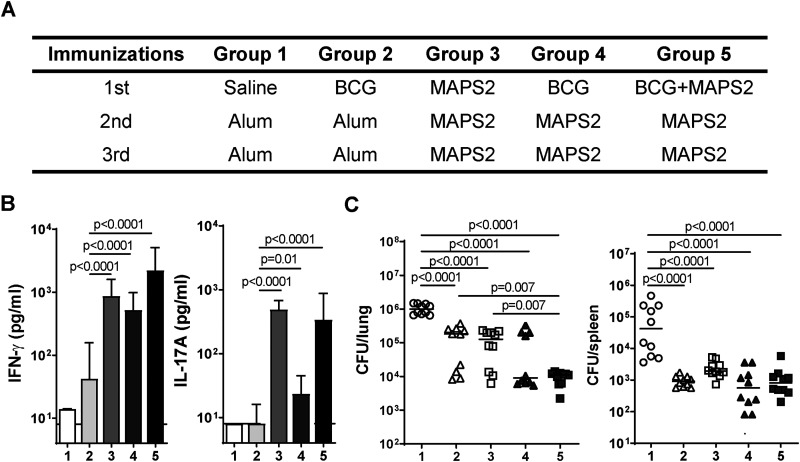
Combined vaccination with MAPS2 and BCG provides enhanced protection against Mtb lung infection compared with either vaccine used alone. (A) Five groups of mice (*n* = 10 per group) received three immunizations with reagents indicated in the table. (B) Peripheral blood was taken 2 weeks after the last immunization and stimulated with Mtb lysate for 6 days. IFN-γ and IL-17A levels in culture supernatants were measured by ELISA. (C) Two weeks after bleeding (1 month after the last immunization), mice were infected with Mtb. One month later, lungs and spleens were harvested for CFU enumeration. Bars indicate medians plus 95% confidence intervals. Lines indicate medians. Dotted lines represent the lower detection limit.

To evaluate whether protection could be enhanced with the addition of another antigen, MPT51 (38, 39), and to simplify the manufacture, we formulated MAPS2 vaccine with three rhavi-fusion constructs (Table 1, MAPS2). Immunization of mice with MAPS2 induced strong antibodies and cellular responses to the first six antigens and specific responses to MPT51 (see Fig. S1 in the supplemental material). The efficacy of MAPS2 was further evaluated and compared to that of BCG in the aerosol infection model, this time with two mouse groups that received both BCG and MAPS2, administered sequentially (group 4) or simultaneously (group 5), to see if a synergistic effect of the two vaccines could be observed (Fig. 2A). Vaccine-induced antigen-specific cellular responses were analyzed 2 weeks after the last immunization by stimulating peripheral blood samples with a whole-cell lysate of Mtb. Upon stimulation, blood cells from BCG-vaccinated mice produced a moderate level of IFN-γ but minimal IL-17A, whereas cells from MAPS2-vaccinated mice responded with production of both IFN-γ and IL-17A (Fig. 2B, group 2 and group 3). Interestingly, immunization with MAPS2 either after or simultaneously (at a separate site) with BCG significantly enhanced IFN-γ- and, particularly, IL-17A-associated cellular responses compared to BCG alone (Fig. 2B, group 4 and group 5 versus group 2; for IFN-γ, *P* < 0.0001 for both groups; for IL-17A, *P* = 0.0141 or *P* < 0.0001, respectively). Two weeks after blood collection, mice were infected with the Mtb strain H37Rv, and lungs and spleens were harvested 1 month later. Consistent with our findings with MAPS1, MAPS2, when used alone, showed similar protection as BCG. Both vaccine groups had significantly reduced CFU in lungs (~10-fold, *P* < 0.0001 for both groups) and spleens (~20- to 40-fold, *P* < 0.0001 for both groups) compared to the control group, indicating protection against primary infection and subsequent dissemination (Fig. 2C, groups 1 to 3). Furthermore, we found that the combined use of MAPS2 and BCG was beneficial, particularly at conferring protection in the lung. The group that received concurrent administration of BCG and MAPS2 at the time of first immunization followed subsequently by two MAPS2 boosters had an additional 8-fold reduction in lung CFU compared to either vaccine group, or a total 80-fold reduction compared to controls (Fig. 2C, group 5). The group that received only two MAPS2 boosters following BCG vaccination also had an additional 2.6-fold reduction in lung CFU compared to the BCG group (Fig. 2C, group 4). Although this difference did not quite reach statistical significance in this experiment (*P* = 0.09), the finding was confirmed in a separate experiment where boosting with MAPS2 further reduced lung CFU by 3-fold compared to BCG vaccination alone (*P* < 0.0001) (Fig. S2). There was no significant difference between BCG or BCG+MAPS groups in terms of protection in spleens.

10.1128/mbio.03611-22.1 FIG S1 Immunization with MAPS2 elicits robust antibody and cellular responses to included target Mtb antigens. Mice (*n* = 20 per group) were immunized three times with adjuvant vehicle or MAPS2. Peripheral blood was taken 2 weeks after the last immunization for antibody and cellular response analysis. (A) IgG titers against each Mtb protein. Titers were expressed in arbitrary units compared to a standard serum to each Mtb protein. (B and C) IFN-γ and IL-17A levels in culture supernatants after stimulating blood cells with a mixture of six Mtb proteins (ESAT6/CFP10, TB9.8/TB10.4, MPT64, and MPT83) (2 μg/mL) (B) or with MPT51 (10 μg/mL) (C). Bars indicate medians plus 95% confidence intervals. Dotted lines represent the lower detection limit. Download FIG S1, TIF file, 6.2 MB. Copyright © 2023 O’Hara et al. 2023 O’Hara et al. https://creativecommons.org/licenses/by/4.0/ This content is distributed under the terms of the Creative Commons Attribution 4.0 International license.

10.1128/mbio.03611-22.2 FIG S2 Vaccination of mice with BCG followed by MAPS2 provides enhanced protection against Mtb lung infection compared to BCG alone. Two groups of mice (*n* = 10 per group) received one dose of BCG for the first immunization, followed by adjuvant vehicle (BCG) or two boosters of MAPS2 (BCG/MAPS2). The control group received saline, followed by two doses of adjuvant vehicle (Alum). Two weeks after the last immunization, peripheral blood was taken for cellular response analysis. (A) IFN-γ and IL-17A levels in culture supernatants after stimulating blood cells with Mtb lysate or a mixture of six Mtb proteins (protein mix). (B) Two weeks after bleeding, mice were infected with Mtb. One month later, lungs and spleens were harvested for CFU enumeration. Bars indicate medians plus 95% confidence intervals. Lines indicate medians. Dotted lines represent the lower detection limit. Download FIG S2, TIF file, 5.4 MB. Copyright © 2023 O’Hara et al. 2023 O’Hara et al. https://creativecommons.org/licenses/by/4.0/ This content is distributed under the terms of the Creative Commons Attribution 4.0 International license.

### Immunization with TB-MAPS induces long-lived systemic and tissue-resident CD4^+^ and CD8^+^ memory T cells.

To better understand the immunological basis of TB-MAPS-induced protection, we performed immune cell profiling on MAPS2-vaccinated mice by flow cytometry, focusing on a 6-month time point after the last immunization to assess the longevity of MAPS-induced immune memory. First, we looked at the changes of various cell populations in the systemic (blood and spleen) and respiratory (nasal tissue and lungs) compartments. As shown in Fig. S3, MAPS2-immunized mice had an increased number of CD4^+^ and CD8^+^ T cells in peripheral blood and nasal tissue but no changes in the total number of CD4^+^ and CD8^+^ T cells in the spleens or lungs or the total number of natural killer (NK) cells, NKT cells, or γδT cells in any compartment.

**FIG 3:**
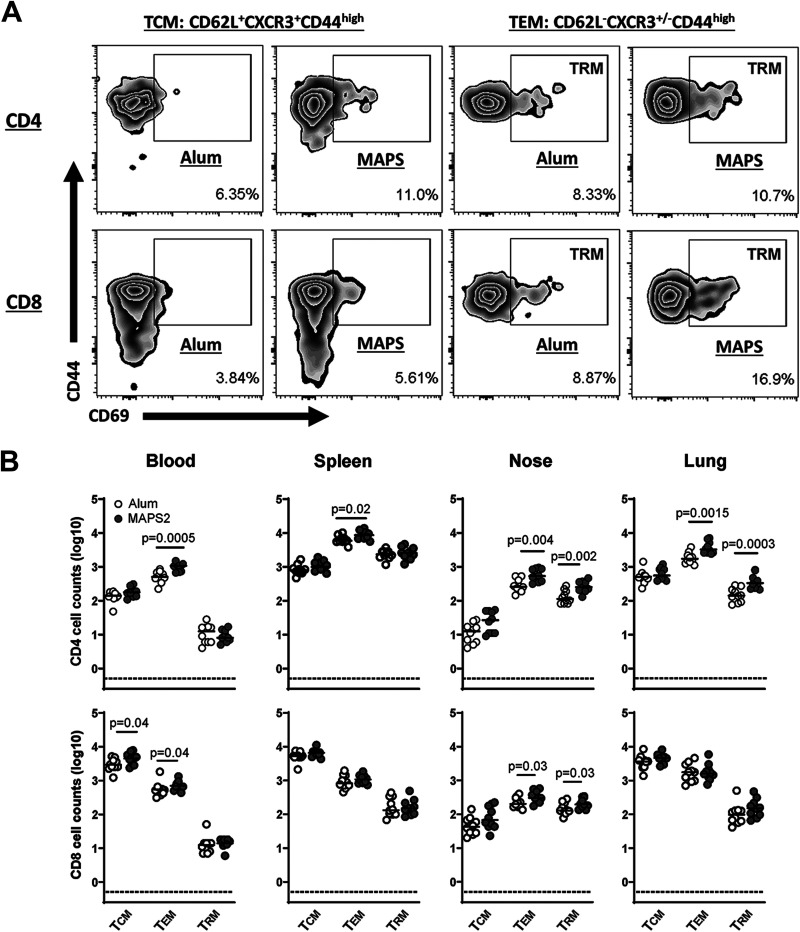
Immunization with MAPS2 induces long-lived systemic and tissue-resident T memory cells. Mice (*n* = 10 per group) were immunized three times with adjuvant vehicle (Alum) or MAPS2. Six months later, blood, spleens, nasal tissue, and lungs were collected, and different populations of CD4^+^ and CD8^+^ T memory cells were quantitated by flow cytometry. (A) Representative zebra plot of the frequency of CD4^+^ or CD8^+^ T_CM_ (CD62L^+^ CXCR3^+^ CD44^high^), T_EM_ (CD62L^−^ CD44^high^), or T_RM_ (CD62L^−^ CD44^high^/CD69^+^) in the lung of Alum- or MAPS2-immunized mice. (B) Absolute counts of CD4^+^ or CD8^+^ T_CM_, T_EM_, and T_RM_ in 100 μL of blood, 1/80 of total splenocytes, 1/3 of total nasal cells, and 1/5 of total lung cells (including both lobes) isolated from Alum- (open circles) or MAPS2-immunized mice (gray circles). *n* = 5 mice per group per analysis. The data represent a summary of two individual analyses. Lines indicate medians. Dotted lines represent the lower detection limit.

10.1128/mbio.03611-22.3 FIG S3 Immune cell profiling of mice after immunization with MAPS2. Mice were immunized three times with Alum or MAPS2 as described in the legend to Fig. 3. Six months later, blood, spleens, nasal tissue, and lungs were collected for cell isolation and flow cytometry analysis. The data show the absolute counts of NK cells (CD3^−^ NK1.1^+^), γδT cells (CD3^+^ TCRβ^−^ NK1.1^−^), CD4^+^ T cells (CD3^+^ TCRβ^+^ NK1.1^−^ CD4^+^ CD8^−^), CD8^+^ T cells (CD3^+^ TCRβ^+^ NK1.1^−^ CD4^−^ CD8^+^), and NKT cells (CD3^+^ TCRβ^+^ NK1.1^+^) in 100 μL of blood, 1/80 of total splenocytes, 1/5 of total lung cells (including both lobes), or 1/3 of total nasal cells isolated from Alum (open circles)- or MAPS2 (gray circles)-immunized mice. *n* = 5 mice per group per analysis. The data represent a summary of two individual analyses. Lines indicate medians. Dotted lines represent the lower detection limit. Download FIG S3, TIF file, 7.6 MB. Copyright © 2023 O’Hara et al. 2023 O’Hara et al. https://creativecommons.org/licenses/by/4.0/ This content is distributed under the terms of the Creative Commons Attribution 4.0 International license.

Next, we examined changes in memory T cell populations. Three memory T cell populations were evaluated: central memory cells (T_CM_, CD62L^+^ CXCR3^+^ CD44^high^), effector memory cells (T_EM_, CD62L^−^ CD44^high^), and tissue-resident effector memory cells (T_RM_, CD69^+^ CD62L^−^ CD44^high^) (Fig. 3A). First, we looked at the peripheral blood and splenic compartments. Compared to control mice, MAPS2-vaccinated mice had significantly increased CD4^+^ T_EM_ and CD8^+^ T_CM_ and T_EM_ in peripheral blood and CD4^+^ T_EM_ in spleen. There was no change in the T_RM_ population in the systemic compartments. In contrast, in the respiratory tissues (nasal passages and lungs), MAPS2 vaccination led to increases not only in total T_EM_ but also in T_RM_. In nasal tissues, this increase was apparent for both CD4^+^ and CD8^+^ T cells, whereas in the lung, it was primarily noted for CD4^+^ T cells.

### Immunization with TB-MAPS induces multipronged antigen-specific memory or memory-like responses in both conventional and unconventional T cells.

**FIG 4:**
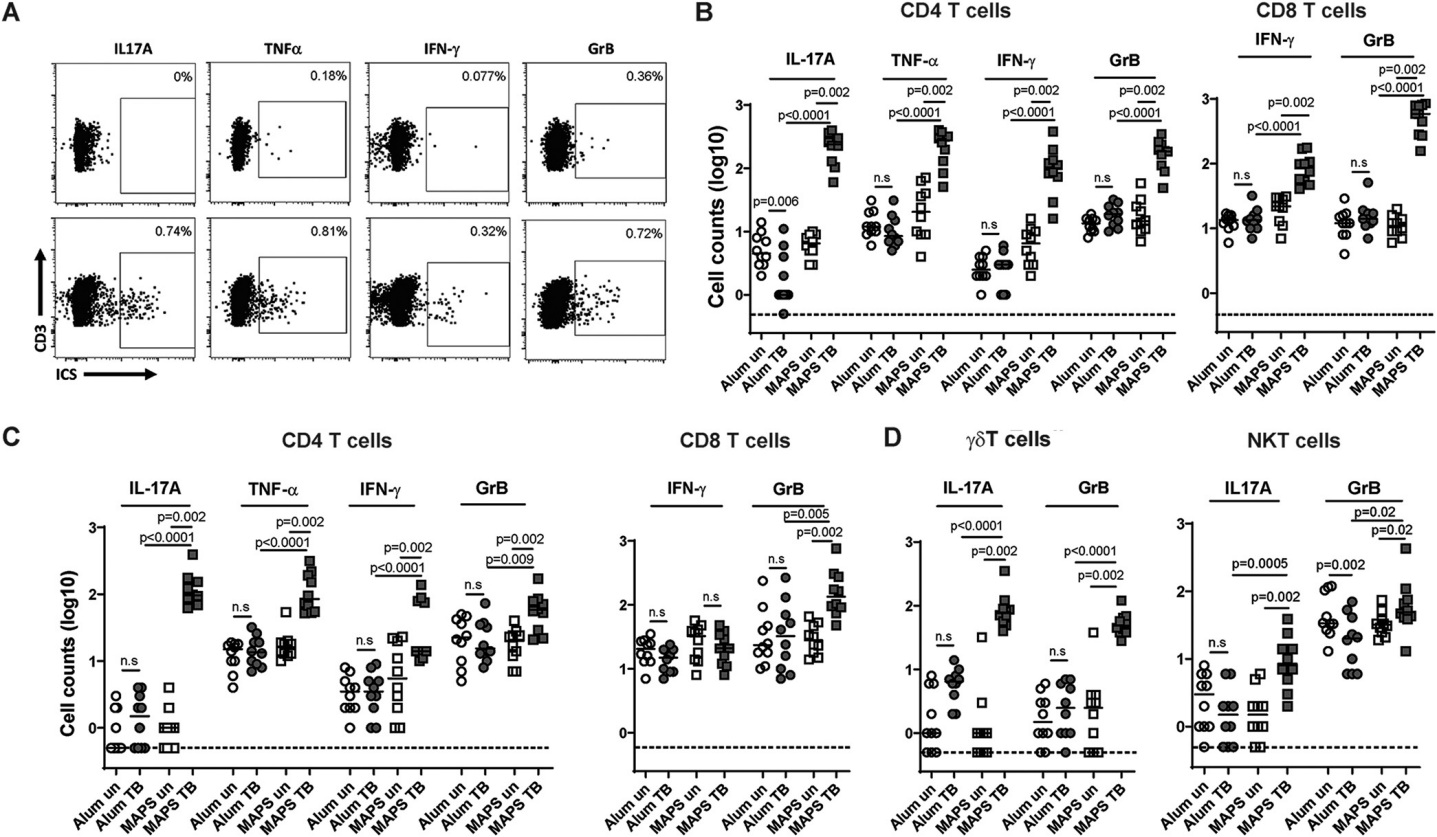
Immunization with MAPS2 leads to differentiation and expansion of a diversity of functional T cells in spleens and lungs. Mice were immunized three times with Alum or MAPS2 as described in the legend to Fig. 3. Six months later, spleens and lungs were collected, and the cells were left unstimulated (U) or stimulated with a mixture of Mtb proteins (S). The production of cytokines or cytotoxic molecules in different T cell populations was analyzed by flow cytometry. (A) Representative dot plots of lung CD4^+^ T cells of Alum- (Alum TB) or MAPS2-immunized mice (MAPS TB) that produce IL-17A, TNF-α, IFN-γ, or granzyme B (GrB) post-stimulation. (B) Absolute counts of IL-17A-, TNF-α-, IFN-γ-, or GrB-producing CD4^+^ (CD3^+^ TCRβ^+^ NK1.1^−^ CD4^+^ CD8^−^) or CD8^+^ (CD3^+^ TCRβ^+^ NK1.1^−^ CD4^−^ CD8^+^) T cells in 1/80 of total splenocytes isolated from Alum- (circles) or MAPS2-immunized (squares) mice without (un) and with Mtb protein stimulation (TB). (C) Absolute counts of IL-17A-, TNF-α-, IFN-γ-, or GrB-producing CD4^+^ or CD8^+^ T cells in the lung (1/5) of Alum- or MAPS2-immunized mice before and post-stimulation. (D) Absolute counts of IL-17A- or GrB-producing γδT cells (CD3^+^ TCRβ^−^ NK1.1^−^) and NKT cells (CD3^+^ TCRβ^+^ NK1.1^+^) in 1/5 of total lung cells (including both lobes) isolated from Alum- (circles) or MAPS2-immunized (squares) mice without (un) and with Mtb protein stimulation (TB). *n* = 5 mice per group per analysis. The data represents a summary of two individual analyses. Lines indicate medians. Dotted lines represent the lower detection limit. n.s., not significant.

Next, we sought to examine the functionality of those memory cells by flow cytometry with intracellular cytokine staining (ICS) analysis. First, we looked at the systemic responses in splenic CD4^+^ and CD8^+^ T cells. Cells were stimulated with a mixture of all seven Mtb antigens (without the rhizavidin moiety) and then stained with antibodies against surface and functional molecules. As shown in Fig. 4A and B, CD4^+^ and CD8^+^ T cells of control mice had no specific responses to Mtb proteins upon stimulation (Alum U versus Alum S). In contrast, the same stimulation readily activated at least four groups of CD4^+^ T cells of MAPS2-immunized mice, with the production of several functional molecules, including IL-17A (Th17 cells), tumor necrosis factor alpha (TNF-α) (Th1 cells), IFN-γ (Th1 cells), and granzyme B (GrB, cytotoxic CD4^+^ T cells) (Fig. 4A and B, MAPS U versus MAPS S). With few exceptions, most responding CD4^+^ T cells produced only one type of functional molecule upon stimulation (data not shown). Similarly, for CD8^+^ cells, MAPS2 vaccination resulted in differentiation and expansion of two different groups of Mtb-specific cytotoxic T lymphocytes (CTL), which produce IFN-γ or GrB, respectively, upon activation.

In the lung, MAPS2-vaccinated but not control mice had developed Mtb-specific CD4^+^ and CD8^+^ T cells. For CD4^+^ cells, again, there were four functional groups, which produce IL-17A, TNF-α, IFN-γ, or GrB, respectively, upon antigen stimulation. But for cytotoxic CD8^+^ cells, we found only one functional group in the lung, with GrB- but not IFN-γ-producing ability.

In addition to conventional αβT cells (e.g., CD4^+^ and CD8^+^ cells), nonconventional T cells (e.g., γδT and NKT cells), representing about 10% of total T cells (CD3^+^) in the lung, are an essential arm of local innate immune defense. While vaccination with MAPS2 did not change the total number of γδT and NKT cells in the lung (as shown earlier in Fig. S3), the responsiveness and functionality of these cells were increased. We found that in control mice, only a small number of lung γδT cells were able to respond to Mtb antigens during *ex vivo* stimulation and produce the proinflammatory cytokine IL-17A (Fig. 4D). In contrast, the number of such Mtb-responsive, IL-17A-producing γδT cells was ~10-fold higher after MAPS2 vaccination (median = 48.5 versus 6.5 in the control group). Furthermore, MAPS2 vaccination also induced the differentiation of another subgroup of γδT cells, characterized by a cytotoxic (rather than proinflammatory) activity, i.e., GrB generating, during stimulation (median = 76.5 versus 2.5 in the control group). A similar picture was seen with NKT cells, which were nonresponsive to Mtb antigens in control mice but became readily activated in MAPS2-vaccinated mice to produce proinflammatory (IL-17A) (median = 8.5 versus 1.5 in the control group) or cytotoxic (GrB) molecules (median = 49.5 versus 20 in the control group).

Due to the limited number of cells isolated from the nasal tissues, we were unable to perform ICS analysis. Instead, we measured cytokine production by enzyme-linked immunosorbent assay (ELISA) after stimulating nasopharyngeal cells with Mtb antigens for 5 days. Consistent with what we found with splenocytes and lung cells, only nasopharyngeal cells from MAPS2-immunized mice but not control mice could respond to Mtb antigens. In the culture supernatant, we detected a substantial amount of IL-17A but not IFN-γ (Fig. S4). Other functional molecules were not analyzed in this experiment due to the limited sample volume. This result confirmed that MAPS2 vaccination indeed induced antigen-specific memory cells in the nasal tissues, although the types of cells need to be further identified.

10.1128/mbio.03611-22.4 FIG S4 MAPS2-induced Mtb-specific IL-17 responses in nasal tissues. Mice were immunized three times with Alum or MAPS2 as described in the legend to Fig. 3. Six months later, nasal tissue was collected for cell isolation and *ex vivo* stimulation with a mixture of 7 Mtb proteins. IL-17A levels in culture supernatants poststimulation were measured by ELISA. *n* = 5 mice per group per analysis. The data represent a summary of two individual analyses. Lines indicate medians. Dotted lines represent the lower detection limit. Download FIG S4, TIF file, 3.7 MB. Copyright © 2023 O’Hara et al. 2023 O’Hara et al. https://creativecommons.org/licenses/by/4.0/ This content is distributed under the terms of the Creative Commons Attribution 4.0 International license.

### MAPS2-induced anti-Mtb immunity is partially dependent on the IL-12p40 signaling pathway.

**FIG 5:**
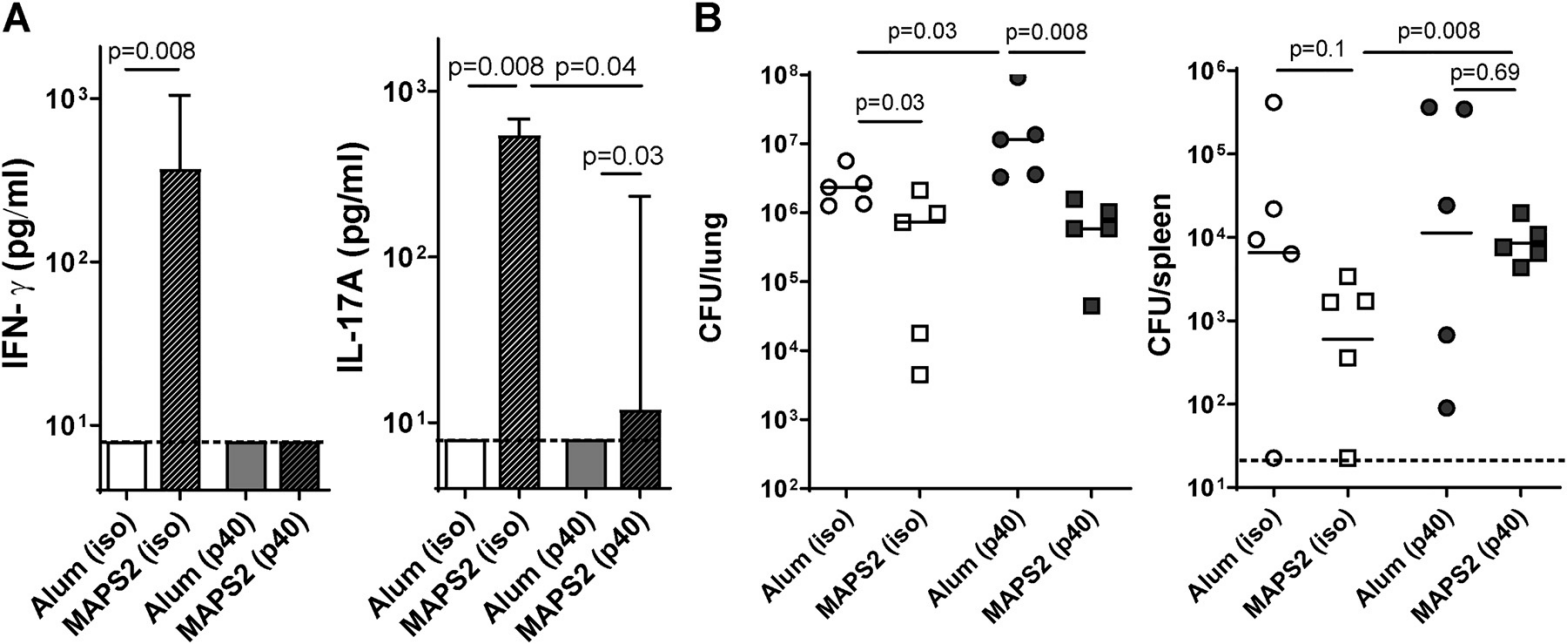
Blocking IL-12p40 signaling differentially impacts MAPS2-induced IFN-γ or IL-17 responses and protection against Mtb lung infection or dissemination. To block IL-12p40 signaling, mice (*n* = 5 per group) received 500 μg anti-IL-12p40 antibody (p40) 1 day before and 3 days after each vaccination with Alum or MAPS2. The control groups received 500 μg isotype control antibody (iso). Mice received three vaccinations in total. Two weeks after the last immunization, peripheral blood was taken for cellular response analysis. (A) IFN-γ and IL-17A levels in culture supernatants after stimulation of whole blood with a mixture of Mtb proteins. (B) Two weeks after bleeding, mice were infected with Mtb. One month later, lungs and spleens were harvested for CFU enumeration. Bars indicate medians plus 95% confidence interval. Lines indicate medians. Dashed lines represent the lower detection limit.

To further understand the protection mechanism at the molecular level, we investigated the impact of the IL-12p40 signaling pathway on MAPS2-induced immune responses and protection. IL-12p40 is the common core subunit of two important cytokines, IL-12 and IL-23, which regulate the differentiation of Th1 cells and IFN-γ-producing CTL (40) or Th17 cells (41), respectively. To block IL-12p40-related signaling (i.e., both IL-12 and IL-23 signaling) during vaccination, we treated mice with anti-IL-12p40 (p40) antibody or an isotype control antibody (iso) 1 day before and 3 days after each immunization. Mice received a total of three immunizations with MAPS2 or adjuvant alone. Vaccine-induced IFN-γ- or IL-17A-associated cellular responses were examined 2 weeks after the last immunization. As shown in Fig. 5A, with isotype control antibody, mice developed strong Mtb-specific responses after MAPS2 vaccination [Fig. 5A, Alum (iso) and MAPS2 (iso)]. Such responses were significantly impaired in mice treated with anti-IL-12p40 antibody [Fig. 5A, Alum (p40) and MAPS2 (p40)]. No IFN-γ was detected after stimulation of blood cells, indicating abrogation of systemic Th1 and IFN-γ CTL responses. In contrast, IL-17A-associated responses were only partially affected.

Following challenge with Mtb, MAPS2 vaccination led to reduced Mtb load in the lungs and, to a lesser extent, in the spleen (dissemination) [Fig. 5B, Alum (iso) versus MAPS2 (iso)] in mice that received isotype control antibody. Treatment with anti-IL-12p40 antibody had a different impact on Mtb lung infection than on blood dissemination. For lung infection, treatment with anti-IL-12p40 antibody significantly increased the bacterial load in the Alum group [Fig. 5B, left panel, Alum (p40) versus Alum (iso)] but did not interfere with protection mediated by MAPS2 [Fig. 5B, left panel, MAPS (p40) versus Alum (p40)]. In contrast, while neutralization of IL-12p40 did not change the ability of Mtb to disseminate in the bloodstream of naive animals [Fig. 5B, right panel, Alum (p40) versus Alum (iso)], this treatment abrogated any protective effect against dissemination following immunization with MAPS2 [Fig. 5B, right panel, MAPS (p40) versus Alum (p40)].

## DISCUSSION

Developing affordable and efficacious vaccines against TB remains an elusive goal. The task is all the more daunting due to the complicated pathogenesis in humans and an incomplete understanding of the nature of protective immunity to Mtb. Most vaccine candidates to date focus on generating Th1 and, to a lesser extent, CD8^+^ cytotoxic responses (5–9), the most-studied anti-Mtb responses previously. However, recent progress in T cell biology and mycobacterial immunology has suggested the critical role of several other T cell subsets, including Th17 (14–16), γδT cells (19–22), and NKT cells (26–28), in fighting Mtb infections, especially for pulmonary disease. Additionally, the discovery of tissue-resident (CD8^+^ and then CD4^+^) memory T cells and their roles in facilitating effective local defense (29–31) also brings new insight into TB vaccine development.

Here, we study a novel vaccine candidate using a platform technology that enables the generation of robust systemic and tissue-resident T cell responses. The TB-MAPS vaccine evaluated here comprises highly defined, purified antigens, thus potentially presenting a lower risk of causing severe adverse effects than whole-cell or virus-vectored vaccines. At the same time, compared to conventional subunit vaccines, TB-MAPS is significantly more immunogenic and able to induce robust antigen-specific humoral and cellular responses (32). The modular nature of the MAPS system offers the flexibility to adjust antigen formulation; in the prototype TB-MAPS vaccines studied here, we included up to seven Mtb proteins to provide an extended antigen coverage, which could be readily modified if needed.

We show here that TB-MAPS induces Mtb-specific, long-lasting memory cells in both systemic and respiratory compartments. MAPS vaccine can activate various types of T cells, including Th1, Th17, cytotoxic CD4^+^ and CD8^+^ cells, γδT cells, and NKT cells, resulting in a broad network of cellular defense. Mice immunized with TB-MAPS demonstrate an increase in the number of systemic T_EM_ in the peripheral circulation, spleen, nasal tissue, and lungs (Fig. 3). Furthermore, the number of T_RM_ in the nasal tissue and lungs (Fig. 3) is also increased, which may enhance protection at the site of initial infection. Third, TB-MAPS induces well-balanced proinflammatory (IL-17- or TNF-α-producing) and cytotoxic (IFN-γ- or GrB-producing) CD4^+^ and CD8^+^ memory cells (Fig. 4) and thus may avoid unnecessary tissue damage due to polarized IFN-γ or IL-17 production (42–46). Lastly, TB-MAPS also induces the differentiation, expansion, and/or migration of nonconventional T cells (γδT and NKT cells) in the lungs (Fig. 4D), which may provide an enhanced (trained) local innate defense. Compared to their naive counterparts, the “trained” γδT cells or NKT cells are more readily activated upon antigen stimulation to produce relevant functional molecules.

Importantly, in an aerosol Mtb infection model, TB-MAPS protects mice from both primary lung infection and subsequent blood dissemination (Fig. 1 and 2). By itself, MAPS-induced protection closely resembles that of BCG, but this protection is enhanced when both BCG and TB-MAPS are given together (at separate sites) or sequentially (Fig. 2 and see also Fig. S2 in the supplemental material). Since BCG is routinely given to infants and young babies with proven efficacy against extrapulmonary disease, a rational approach would be to incorporate any new vaccine in a schedule that includes BCG. Therefore, one approach would be to study the addition of TB-MAPS in routine immunization schedules, as an adjunct to BCG, in infants. Such a strategy would be attractive as it may provide synergistic protective effects derived from both vaccines and perhaps provide protection against pulmonary TB, particularly in adolescents and adults as a boosting dose, which may have the largest impact on transmission.

From a mechanistic standpoint, our results suggest that different types of cellular responses may play diverse roles in vaccine-induced protection against Mtb infection. Blocking IL-12p40 signaling during immunization had a great impact on MAPS-induced IFN-γ responses and protection against Mtb blood dissemination but less so on IL-17 responses or pulmonary protection (Fig. 5). These findings have several implications. First, these results are consistent with the view, supported by the literature (44, 47, 48), that IFN-γ responses are critical in controlling extrapulmonary Mtb. Second, our data suggest that systemic IFN-γ responses, as measured here by *ex vivo* stimulation of peripheral blood samples, are not essential for protection against lung infection. Finally, our results indicate that MAPS can induce IL-17A responses in both an IL-12p40 (or IL-23)-dependent and -independent manner, and the latter may be mediated by γδT cells and/or NKT cells rather than Th17 cells. Compared to IFN-γ responses, IL-17A responses may play an important role in lung protection. This hypothesis could explain why BCG vaccination, which induces strong IFN-γ, but far weaker IL-17A, responses (Fig. 2) (49), has demonstrable protection against disseminated Mtb infection in infants and children but less so against primary pulmonary TB. Moreover, the use of MAPS vaccine in combination with BCG would overcome this problem, i.e., to induce enhanced IL-17A responses, and improve the protection in the lungs (Fig. 2). Future studies will be conducted to dissect further the contribution of individual T cell subsets to MAPS-mediated protection and understand the dynamics and roles of systemic and tissue-resident memory T cells during Mtb infection.

In summary, we demonstrate here that the TB-MAPS vaccine can induce robust and multipronged cellular responses against Mtb antigens. This vaccine construct activates both conventional and nonconventional T cells, resulting in long-lived memory cells/trained innate immunity residing in both systemic and respiratory compartments. MAPS vaccination provides significant protection against Mtb lung infection and dissemination and can further enhance the efficacy of BCG when the two vaccines are used together. These properties provide strong support for further preclinical and clinical development of TB-MAPS as a preventative vaccine against TB.

## MATERIALS AND METHODS

### Mouse and bacterial strains.

Wild-type C57BL/6 female mice were purchased from Jackson Laboratory (Bar Harbor, ME) and housed under specific-pathogen-free (SPF) animal biosafety level 2 (ABSL2) conditions for all immunizations and immunogenicity studies (Boston Children’s Hospital) and transferred to an ABSL3 facility (Harvard Center for Comparative Medicine) for Mtb infections. Mtb strain H37Rv was kindly provided by BEI Resources (managed by American Type Culture Collection [ATCC]). Escherichia coli strains DH5α, BL21(DE3), and T7 shuffle express were purchased from New England Biolabs (NEB; Ipswich, MA).

### Ethics statement.

All procedures involving mice were approved by the animal care and use committee at Boston Children’s Hospital (IACUC protocol number 191040512) or Harvard Medical Area (HMA) (IACUC protocol number 03000), following the National Institutes of Health guidelines for animal housing and care.

### Cloning and purification of Mtb antigens and lipidated rhizavidin.

DNA sequences encoding ESAT6, CFP10 (fragment 1–41 and fragment 45–80), TB9.8, TB10.4, MPT64 (25–228), MPT83 (58–220), and MPT51 (33–299) were amplified from Mtb genomic DNA (H37Rv strain) (a kind gift of Robert Husson, Boston Children’s Hospital) by conventional PCR and cloned into the pET21b vector for recombinant expression in E. coli as described previously (32). For rhavi fusion proteins, seven constructs, each of which contained up to three Mtb antigens, were prepared by inserting Mtb DNA sequence(s) at the 3′ end of the rhavi gene (see Table S1 in the supplemental material) and then cloned into the pET21b vector. Lipidated rhavi was constructed by adding a lipidation box at the 5′ end of the rhavi gene as described previously (32). All Mtb proteins (nonfusion) and lipidated rhavi were expressed in E. coli BL21(DE3) cells, and all rhavi-Mtb fusion antigens were expressed in T7 shuffle express cells. The recombinant His-tagged proteins were then purified using nitrilotriacetic acid (NTA) resin followed by size exclusion chromatography as described previously (32). The purified proteins were then concentrated, filtered via 0.2-μm filters, aliquoted, and stored at −80°C until use.

10.1128/mbio.03611-22.8 TABLE S1 List of rhavi-Mtb fusion proteins. Download Table S1, DOCX file, 0.02 MB. Copyright © 2023 O’Hara et al. 2023 O’Hara et al. https://creativecommons.org/licenses/by/4.0/ This content is distributed under the terms of the Creative Commons Attribution 4.0 International license.

### Preparation of MAPS complexes.

MAPS complexes were prepared as described previously (32). Briefly, type 1 pneumococcal capsular polysaccharide (CPS1) was purchased from ATCC and then biotinylated using 1-cyano-4-dimethylaminopyridinium tetrafluoroborate (CDAP) as the activation reagent. The MAPS complex was assembled by incubation of biotinylated CPS1 with lipidated rhavi or individual Mtb rhavi fusion proteins at room temperature overnight. The input ratio of protein to polysaccharide was 3:1 (wt/wt). The assembled complex was isolated by size exclusion chromatography. The fractions containing the MAPS complex were pooled and concentrated by ultrafiltration. The protein concentration in each MAPS complex was measured using a bicinchoninic acid (BCA) protein assay kit (Pierce). The incorporation of target antigens onto CPS1 was examined on a reduced SDS-PAGE gel.

### Immunization.

All TB-MAPS vaccines were formulated the day prior to immunization. MAPS complexes were diluted to the appropriate concentration in saline and then mixed with aluminum hydroxide (Alum) (Brenntag) (1.25-mg/mL final concentration) in 5-mL tubes and incubated at 4°C overnight with rotation (24 rpm). Six-week-old female mice (*n* = 10) received subcutaneous immunizations on the upper back, once every other week for a total of 3 injections. For MAPS1, mice received 10 μg of each Mtb complex and 5 μg of lipidated rhavi complex per dose per mouse. For MAPS2, mice received 12.5 μg of rhavi-ESAT6/CFP10-MPT64 complex and rhavi-TB9.8/TB10.4-MPT83 complex, 5 μg of rhavi-MPT51 complex, and 2.5 μg of lipidated rhavi complex per dose per mouse. The control groups received Alum (1.25-mg/mL final concentration in saline) alone. For BCG immunization, mice received one subcutaneous injection of 200 μL of BCG vaccine (Merck, diluted to 1 × 10^5^ CFU in 200 μL with saline just before immunization). The control group received 200 μL of saline. For BCG followed by MAPS2, mice received one subcutaneous injection with BCG and, 1 month later, received two boosters of MAPS2 at a 2-week interval. The control group received BCG followed by Alum. For BCG and MAPS2 coadministration, mice received one injection of BCG and one injection of MAPS2 at opposite flanks for the first immunization and, 1 month later, received two boosters of MAPS2 at a 2-week interval. For IL-12p40 neutralization studies, mice received 500 μg anti-IL-12p40 antibody (clone 17.8; BioXcell, Lebanon, NH) or isotype control antibody (clone 2A3; BioXcell, Lebanon, NH) via intraperitoneal injection 1 day before and 3 days after each immunization.

### Mtb culture and infection.

Mtb strain H37Rv was used for aerosol infection in mice. Bacteria were cultured in Middlebrook 7H9 containing 0.2% (vol/vol) glycerol, 15 mM NaCl, 0.05% (vol/vol) Tween 80, and 10% (vol/vol) oleic acid-albumin-dextrose-catalase (OADC) supplement (Fisher Scientific, Waltham, MA) and maintained at 37°C with shaking at 100 rpm until log-phase growth. Cells were then sonicated and diluted in phosphate-buffered saline (PBS) to 1 × 10^6^ CFU/mL for inoculation in mice.

Mice were infected with aerosolized Mtb using a Glas-Col aerosol chamber (50), attached with a Venturi nebulizer unit filled with 1 × 10^6^-CFU/mL Mtb solution to achieve an estimated infection dose of ~100 CFU/mouse. In each experiment, five age-matched unvaccinated mice were included to enumerate bacterial deposition in the lung 24 h postinfection. Lungs were harvested in PBS and homogenized using a stomacher homogenizer under BSL3 conditions. Samples were diluted in PBS and plated on 7H10 plates supplemented with OADC and cycloheximide and incubated at 37°C for 3 weeks. On average, 75 to 80 CFU of Mtb was recovered from the lungs of naive mice 24 h after infection. One month after infection, immunized mice were sacrificed by isoflurane overdose, and lungs and spleens were harvested, homogenized, and plated for CFU enumeration. For plates that had no visible colonies, a value of 0.8 CFU/organ (lower detection limit) was assigned.

### Antibody and cytokine analysis.

Two weeks after the last immunization, animals were bled using heparin-lithium-coated tubes (BD Bioscience) under isoflurane anesthesia for antibody and cellular response analysis. Antigen-specific IgG antibody was measured by ELISA. Immulon 2 HB 96-microwell plates (Thermo Scientific) were coated with 1 μg/mL of individual recombinant Mtb protein (without rhavi protein) in PBS at room temperature overnight. The plates were washed with PBS containing 0.05% Tween 20 (PBS-T) and then blocked with 1% bovine serum albumin (BSA) in PBS for 1 h. After blocking, serial dilutions of mouse plasma were added and incubated for 2 h, followed by a 1-h incubation with horseradish peroxidase (HRP)-conjugated secondary antibody against mouse IgG. The plates were then washed and developed with SureBlue TMB microwell peroxidase substrate (SeraCare Life Sciences, Milford, MA). A 1 M concentration of HCl was used to terminate the reactions before the plate was analyzed for absorbance at 450 nm (*A*_450_) using a spectrophotometer. Antibody titers were analyzed using SoftMax Pro, version 5.3 (Molecular Devices, San Jose, CA), and expressed in arbitrary units relative to a standard serum.

For antigen-specific cellular responses, 25 μL of heparinized blood was added to 225 μL stimulation medium (Dulbecco modified Eagle medium [DMEM] [BioWhittaker Inc., Walkersville, MD]) containing 10% low-endotoxin defined FBS (HyClone, Cytiva, Marlborough, MA), 50 μM 2-mercaptoethanol (Sigma), and 10 μg/mL ciprofloxacin (Cellgro, Lincoln, NE) in sterile 96-well round-bottomed tissue culture plates (Thermo Scientific). The cultures were incubated at 37°C for 6 days in the presence of a mixture of ESAT6/CFP10, TB9.8/TB10.4, MPT64, and MPT83 proteins (equal weight ratio, 2 μg/mL of total proteins), MPT51 protein (10 μg/mL), or an Mtb lysate (10 μg/mL) (NR-14822; BEI Resources). Supernatants were then collected following centrifugation, and IFN-γ and IL-17A concentrations were determined by ELISA using mouse IL-17A or IFN-γ ELISA kits (R&D Systems, Minneapolis, MN). ELISA plates were read at *A*_450_ using a spectrophotometer and analyzed using SoftMax Pro, version 5.3 (Molecular Devices, San Jose, CA). For cellular responses in nasal tissues, nasal cell suspensions in the stimulation medium were aliquoted into 24-well tissue culture plates and stimulated with a mixture of Mtb antigens (ESAT6/CFP10, TB9.8/TB10.4, MPT64, MPT83, and MPT51, equal weight ratio, 1.25 μg/mL of total proteins) for 5 days. Supernatants were then harvested for cytokine analysis by ELISA. A value of 8 pg/mL was set as the lower detection limit for IL-17A or IFN-γ cytokine assay.

### Cell isolation from blood, nasal tissue, lungs, and spleens.

Mice were anesthetized under isoflurane and then exsanguinated via retro-orbital bleed (in heparin-lithium-coated tubes) (BD Bioscience). Three hundred microliters of blood was aliquoted from each mouse and spun at 800 × *g* for 10 min to separate and remove the plasma. The pellet was resuspended in 10 mL of ACK lysis buffer (Lonza Biologics, Portsmouth, NH) in a 50-mL conical tube and incubated at room temperature (RT) for 30 min to lyse red blood cells. The lysis was terminated by adding 30 mL of wash buffer (PBS plus 2% fetal bovine serum [FBS]), and the cells were pelleted by centrifugation at 400 × *g* for 5 min. The cells were then washed once with 10 mL of wash buffer, pelleted, resuspended in 3 mL of tissue culture medium (TCM; 50:50 DMEM–F-12, 10% FBS, 50 mM β-mercaptoethanol, 10 μg/mL ciprofloxacin), and stored on ice until use.

The nasal tissue was isolated and processed as previously described (51). Briefly, the lower jaw and nasal mucosa-associated lymphoid tissue (NALT) were removed before separating the nasal passage from the rest of the head by coronally cutting behind the eyes and before the ear canal. The nasal tissue was disrupted in a petri dish with forceps, washed in Hanks balanced salt solution (HBSS) (Cellgro), and dissociated with 25 mL collagenase solution per animal (collagenase type IV [Thermo Fisher Scientific]; 225 U/mL, 20% FBS, 1.5 mM CaCl_2_ in HBSS) by vigorous shaking at 37°C for 45 min. The dissociated tissue was passed through a 70-μm strainer, washed with 30 mL wash buffer twice, resuspended in 3 mL of TCM, and stored on ice until use.

For lung cells, both lobes of the lungs were dissected and placed into 20 mL of cold HBSS. After vigorously shaking to release blood cells, the lung tissues were put into a petri dish, cut into small pieces by scalpel, and then transferred into 8 mL of collagenase buffer (225 U/mL collagenase type IV, 20% FBS, 1.5 mM CaCl_2_, 20 U/mL DNase, HBSS) for incubation at 37°C for 30 min with shaking (~220 rpm). At the end of incubation, the digestion mixture was transferred into a gentleMACS C tube with 10 mL of wash buffer and homogenized using program m_lung_02_01 on a gentleMACS dissociator (Miltenyi Biotec, Cambridge, MA). After homogenization, the mixture was transferred into a 50-mL tube with 20 mL of wash buffer, and the cells were pelleted by centrifugation at 400 × *g* for 5 min. The cell pellet was then resuspended in 3 mL ACK lysis buffer and incubated at RT for 2 min to lyse red blood cells. The lysis was terminated by adding 30 mL of wash buffer (PBS plus 2% FBS). The cells were pelleted, washed once with 10 mL of wash buffer, passed through a 70-μm strainer, pelleted again, and resuspended in 5 mL of TCM and stored on ice until use.

Splenocytes were isolated as described previously (32). Briefly, spleens were dissected and then disrupted in a petri dish using the plunger of a 3-mL syringe. The cells were resuspended in wash buffer, passed through a 70-μm strainer, pelleted after centrifugation, and then resuspended in 1 mL ACK lysis buffer to remove red blood cells. After lysis, the cells were resuspended in wash buffer, pelleted, washed once with wash buffer, and then resuspended in 2 mL of TCM and stored on ice until use.

### Cell surface immunophenotyping and intracellular cytokine staining by flow cytometry.

**(i) Antibodies and reagents.** Antibodies and reagents from BioLegend (San Diego, CA) included Zombie violet, Fc block, fluorescein isothiocyanate (FITC) anti-CD69 (clone H1.2F3), phycoerythrin (PE) anti-CD4 (clone RM 4-4), peridinin chlorophyll protein (PerCP)/Cy5.5 anti-TCRβ (clone H57-597), PE/Cy7 anti-CD44 (clone IM7), allophycocyanin (APC) anti-CXCR3 (clone CXCR3-173), APC/Cy7 anti-CD8α (clone 53-6.7), BV510 anti-NK1.1 (clone PK136), BV605 anti-CD62L(clone MEL-14), BV711 anti-CD3 (clone 17A2), BV510 anti-CD4 (clone RM4-4), BV605 anti-CD45 (clone 30-F11), PE/Cy7 anti-NK1.1 (clone PK136), BV510 anti-TCRγδ (clone GL3), FITC anti-IL-17A (clone TC11-18H10.1), PE anti-granzyme B (clone QA16A02), and PE/Cy7 anti-TNF-α (clone MP6-XT22). Antibodies and reagents from BD Biosciences included APC anti-IFN-γ (clone XMG1.2), GolgiStop, and the Cytofix/Cytoperm plus kit.

**(ii) Immunophenotyping.** One milliliter of cell suspension from blood, nasal tissue, or lungs or 50 μL of splenocytes was used for each sample. Cells were pelleted and washed with 1 mL of PBS. For surface staining, cells were sequentially incubated with Zombie violet (live/dead signal), Fc block (1 μg/mL), and then a cocktail including FITC anti-CD69, PE anti-CD4, PerCP/Cy5.5 anti-TCRβ, PE/Cy7 anti-CD44, APC anti-CXCR3, APC/Cy7 anti-CD8α, BV510 anti-NK1.1, BV605 anti-CD62L, and BV711 anti-CD3. After staining, cells were washed with staining buffer (SB; PBS with 2% FBS and 0.1% NaN_3_), pelleted, and resuspended in 400 μL of SB. Data were acquired on an Attune NxT flow cytometer (Thermo Fisher Scientific) with a 350-μL sample volume for blood, nasal tissue, and lung cells or a 200-μL sample volume for splenocytes.

**(iii) Intracellular cytokine staining.** One milliliter of cell suspension from lungs or 50 μL of splenocytes was used for each sample. Cells were plated in a 24-well plate (1 mL per well) in TCM in the absence or presence of a mixture of Mtb proteins (ESAT6/CFP10, TB9.8/TB10.4, MPT64, MPT83, and MPT51; 2.5 μg/mL for lung cells, 1.25 μg/mL for splenocytes). After overnight incubation, cells were treated with 1 μg/mL of GolgiStop (BD) for 5 h. Cells were then harvested, stained for surface molecules, and then permeabilized, fixed, and stained for intracellular molecules following the instructions of the Cytofix/Cytoperm Plus kit. For the T cell panel, cells were stained with PerCP/Cy5.5 anti-TCR, APC/Cy7 anti-CD8α, BV510 anti-CD4, BV605 anti-CD45, and BV711 anti-CD3 first and then with FITC anti-IL-17A, PE anti-granzyme B, PE/Cy7 anti-TNF-α, and APC anti-IFN-γ. For the γδT/NKT panel, cells were stained with PerCP/Cy5.5 anti-TCRβ, PE/Cy7 anti-NK1.1, APC/Cy7 anti-CD8α, BV510 anti-TCRγδ, BV605 anti-CD45, and BV711 anti-CD3 first and then with FITC anti-IL-17A, PE anti-granzyme B, and APC anti-IFN-γ. After staining, cells were washed with SB, pelleted, and resuspended in 400 μL of SB. The fluorescence-minus-one control for IL-17A, IFN-γ, TNF-α, or GrB was prepared using poststimulation splenocytes of MAPS2-immunized mice. Data were acquired with a 350-μL sample volume for blood, nasal tissue, and lung cells or a 200-μL sample volume for splenocytes.

Single staining samples were prepared using UltraComp eBeads (Thermo Fisher Scientific) and indicated antibodies following manufacturer’s instructions. All data were analyzed using FlowJo software (TreeStar, version 10). Examples of gating strategy are shown in Fig. S5 to S7.

10.1128/mbio.03611-22.5 FIG S5 Gating strategy—immunophenotyping of lung cells from a MAPS2-immunized mouse. Download FIG S5, TIF file, 0.4 MB. Copyright © 2023 O’Hara et al. 2023 O’Hara et al. https://creativecommons.org/licenses/by/4.0/ This content is distributed under the terms of the Creative Commons Attribution 4.0 International license.

### Statistical analysis.

All statistical analyses were performed using Prism (version 9.4) (GraphPad Software, Inc., San Diego, CA). Antibody titer, cytokine production, CFU in organs, and cell counts were compared between indicated groups using the Mann-Whitney U test. For ICS analysis, the counts of cytokine-producing cells in the same group (Alum or MAPS2) without or with TB antigen stimulation were compared using the Wilcoxon test.

10.1128/mbio.03611-22.6 FIG S6 Gating strategy—intracellular cytokine staining of lung CD4^+^ and CD8^+^ T cells from a MAPS2-immunized mouse. Download FIG S6, TIF file, 0.6 MB. Copyright © 2023 O’Hara et al. 2023 O’Hara et al. https://creativecommons.org/licenses/by/4.0/ This content is distributed under the terms of the Creative Commons Attribution 4.0 International license.

10.1128/mbio.03611-22.7 FIG S7 Gating strategy—intracellular cytokine staining of lung γδT and NKT cells from a MAPS2-immunized mouse. Download FIG S7, TIF file, 0.6 MB. Copyright © 2023 O’Hara et al. 2023 O’Hara et al. https://creativecommons.org/licenses/by/4.0/ This content is distributed under the terms of the Creative Commons Attribution 4.0 International license.
