# Supplementary figures and images for: A MAPS Vaccine Induces Multipronged Systemic and Tissue-Resident Cellular Responses and Protects Mice against Mycobacterium tuberculosis
Source: mBio. 2023 Feb 7;14(1):e03611-22. doi: 10.1128/mbio.03611-22 (PMC9973048; doi:10.1128/mbio.03611-22)

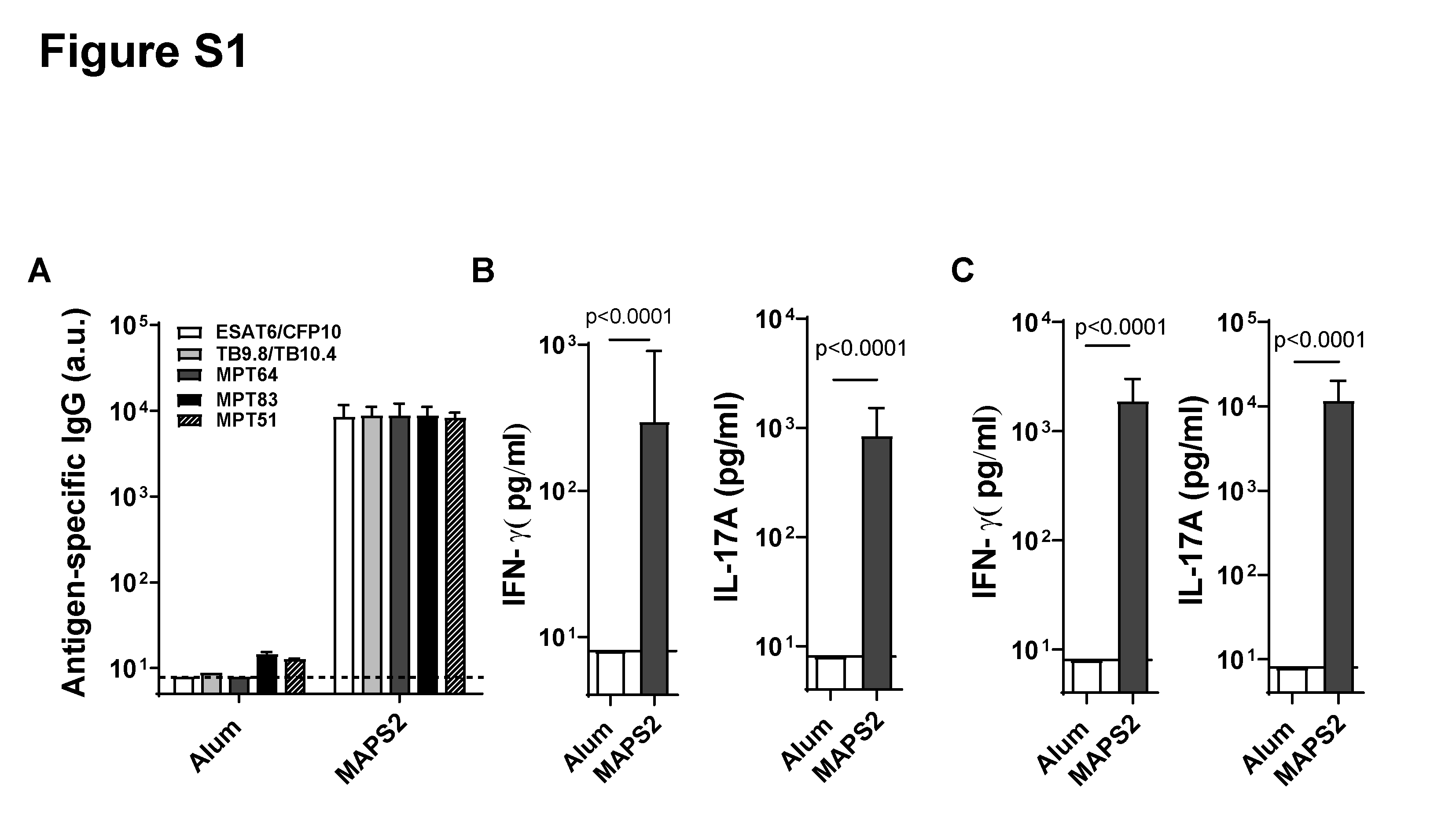

Supplement: FIG S1 [file mbio.03611-22-s0001.tif]

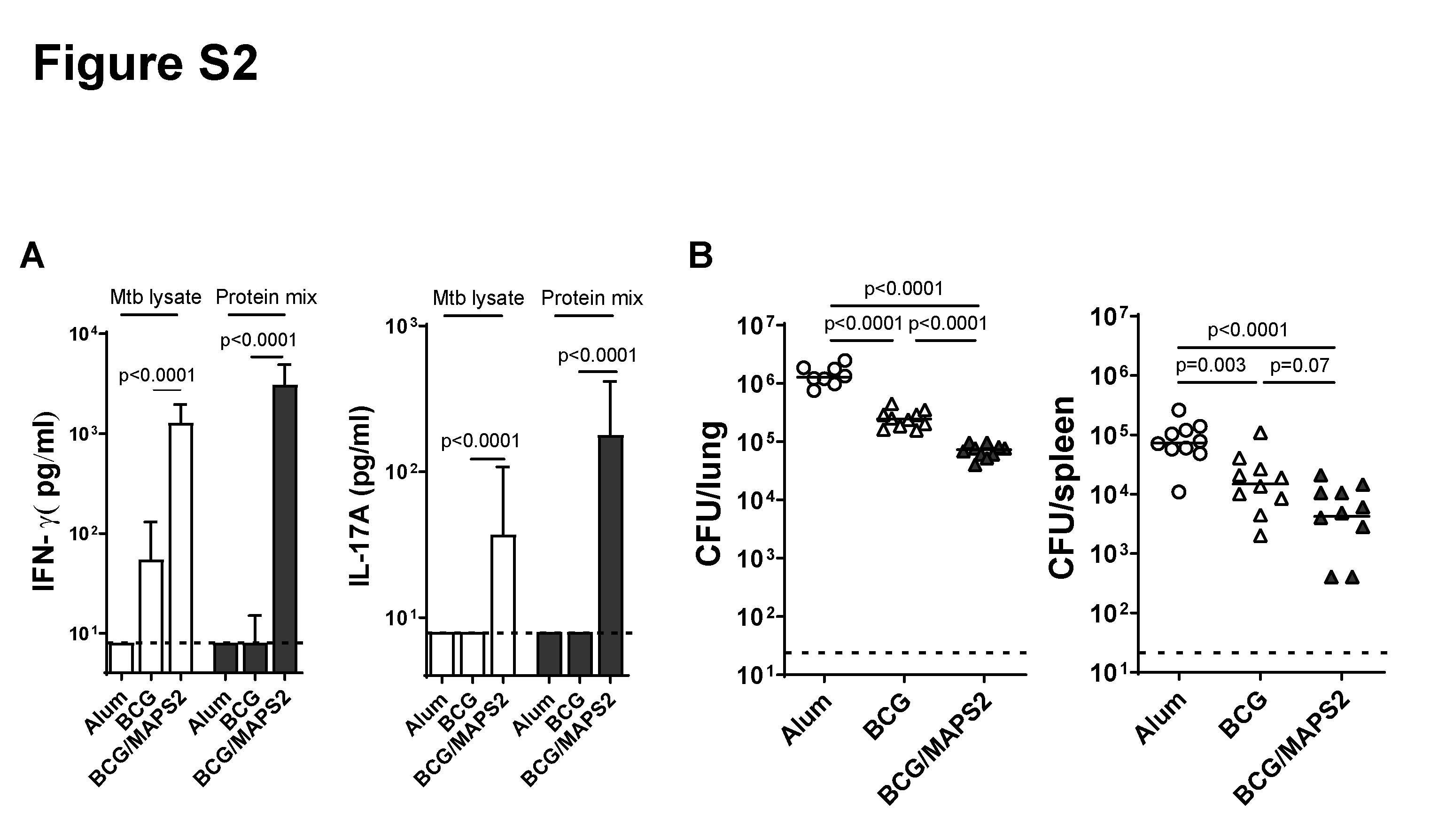

Supplement: FIG S2 [file mbio.03611-22-s0002.tif]

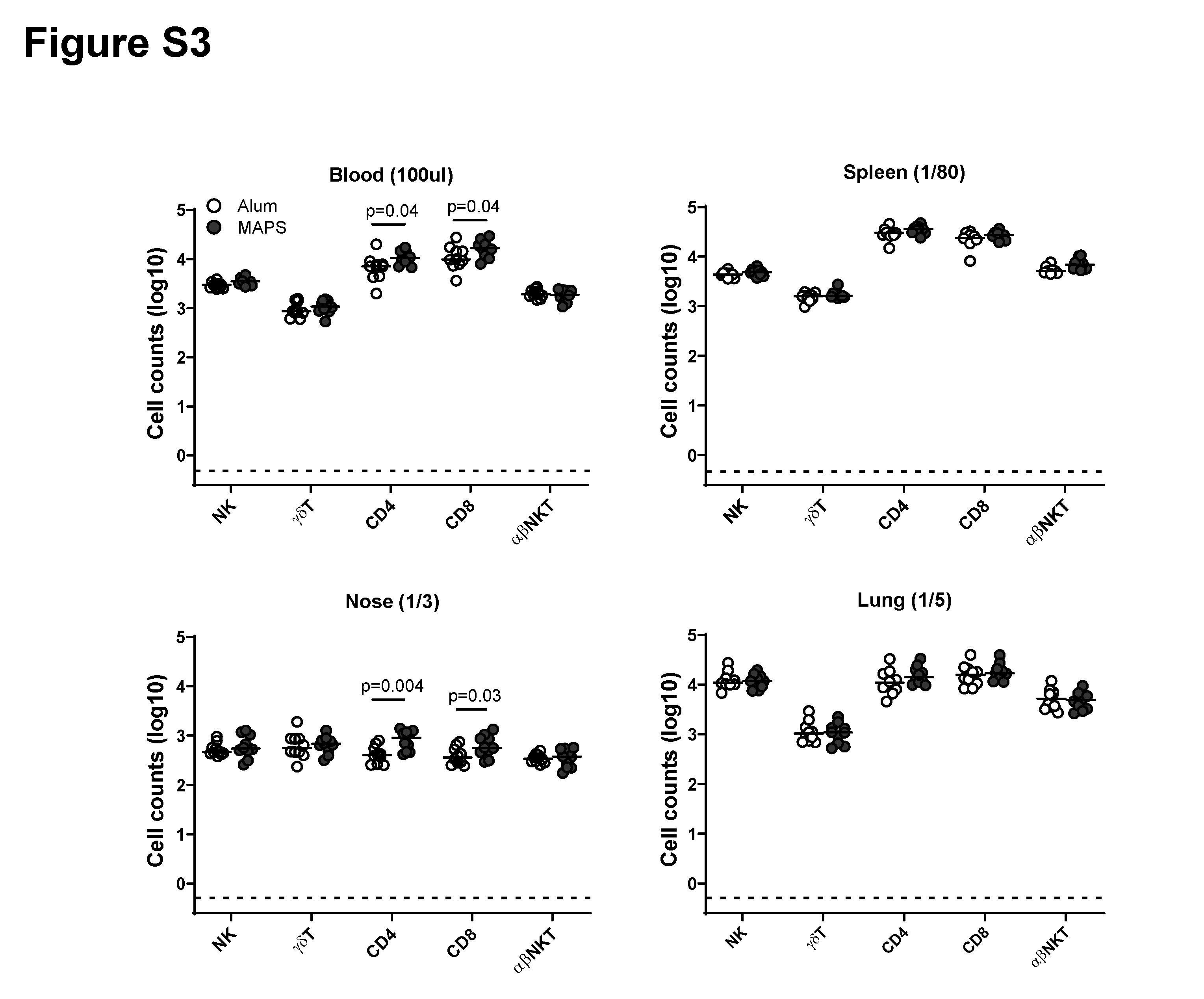

Supplement: FIG S3 [file mbio.03611-22-s0003.tif]

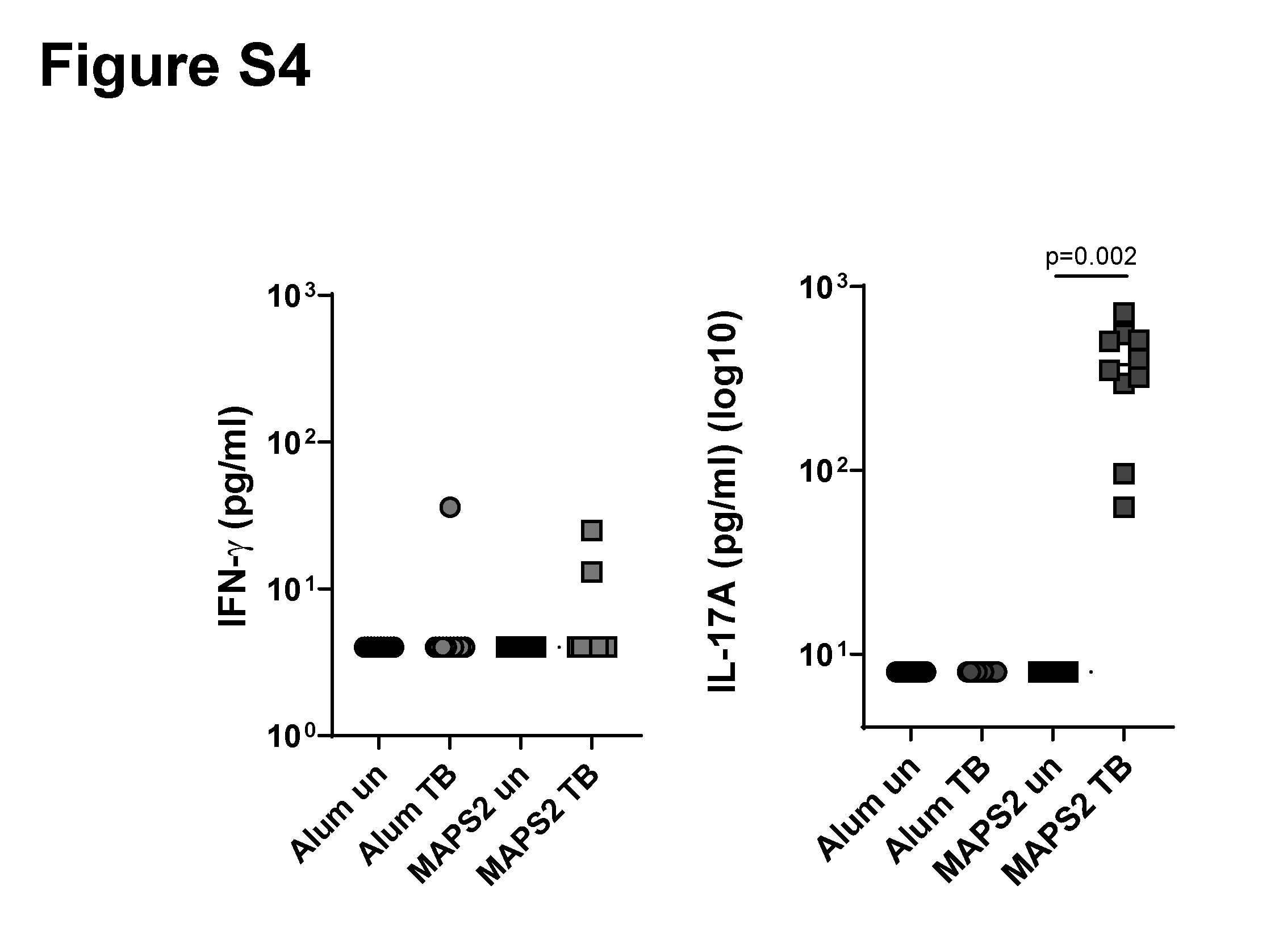

Supplement: FIG S4 [file mbio.03611-22-s0004.tif]

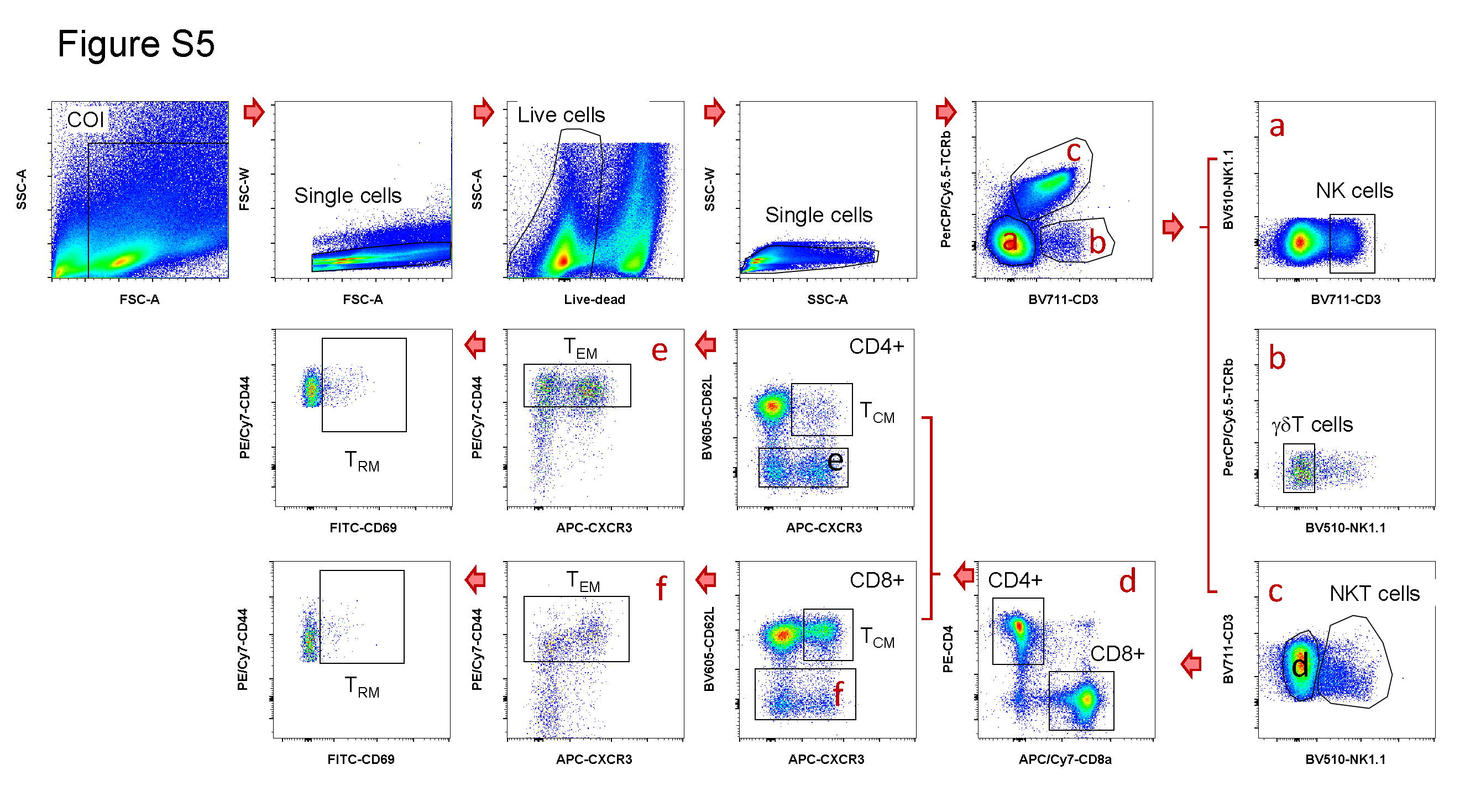

Supplement: FIG S5 [file mbio.03611-22-s0005.tif]

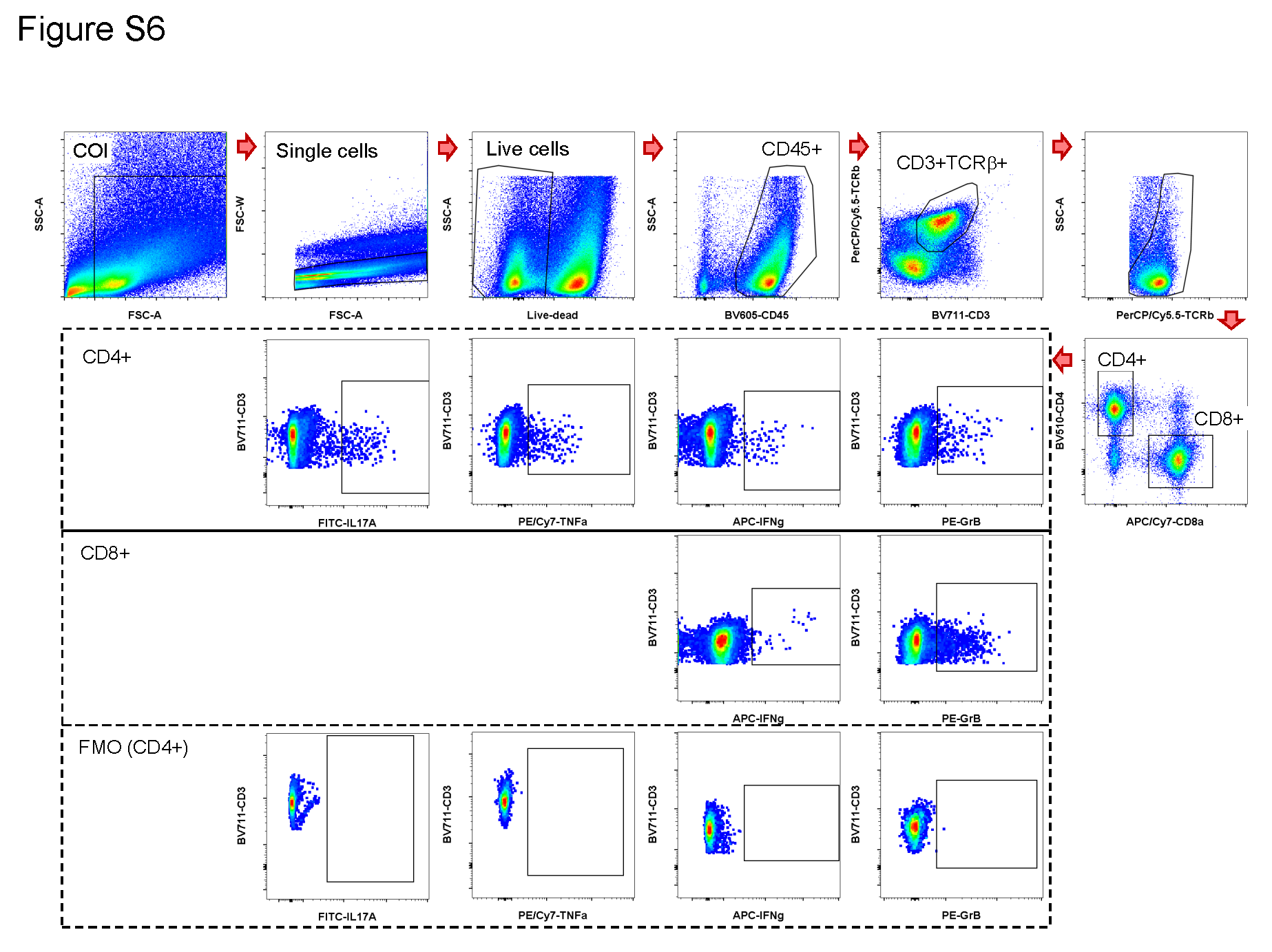

Supplement: FIG S6 [file mbio.03611-22-s0006.tif]

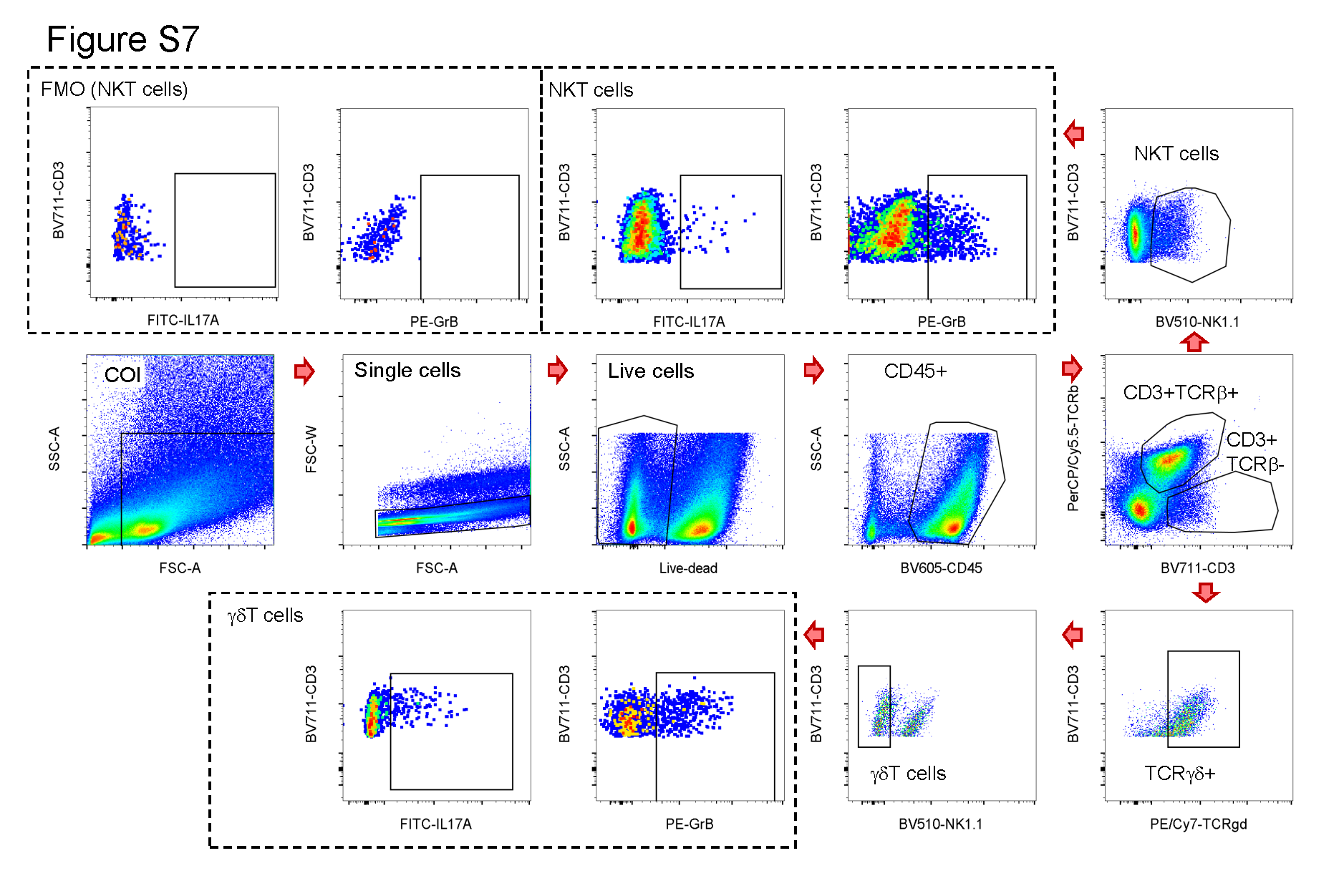

Supplement: FIG S7 [file mbio.03611-22-s0007.tif]
