# Supplementary material for: A MAPS Vaccine Induces Multipronged Systemic and Tissue-Resident Cellular Responses and Protects Mice against Mycobacterium tuberculosis
Source: mBio. 2023 Feb 7;14(1):e03611-22. doi: 10.1128/mbio.03611-22 (PMC9973048; doi:10.1128/mbio.03611-22)
Supplement: TABLE S1 [file mbio.03611-22-s0008.docx]

Table S1

| **Fusion Proteins** | **Constructs*** |
| --- | --- |
| Rhavi-ESAT6/CFP10 | Rhavi-ESAT6/CFP10 (45-80/1-41) |
| Rhavi-TB9.8/TB10.4 | Rhavi-TB9.8/TB10.4 |
| Rhavi-MPT64 | Rhavi-MPT64 (25-228) |
| Rhavi-MPT83 | Rhavi-MPT83 (58-220) |
| Rhavi-MPT51 | Rhavi-MPT51 (33-299) |
| Rhavi-ESAT6/CFP10-MPT64 | Rhavi-ESAT6/CFP10 (45-80, 1-41)-MPT64 (25-228) |
| Rhavi-TB9.8/TB10.4-MPT83 | Rhavi-TB9.8/TB10.4-MPT83 (58-220) |
|  | |
